# Association Between Epicardial Adipose Tissue and Stroke

**DOI:** 10.3389/fcvm.2021.658445

**Published:** 2021-04-21

**Authors:** Maria Inês Rosa, Antonio José Grande, Leticia Dorsa Lima, Eduardo Ronconi Dondossola, Maria Laura Rodrigues Uggioni, Adrian V. Hernandez, Gary Tse, Tong Liu, Octávio Marques Pontes-Neto, Giuseppe Biondi-Zoccai, Mansueto Gomes Neto, André Rodrigues Durães, Michel Pompeu B. O. Sá, Elmiro Santos Resende, Leonardo Roever

**Affiliations:** ^1^Laboratory of Biomedicine Translational, University of Extremo Sul Catarinense, Criciúma, Brazil; ^2^Department of Medicine, State University of Mato Grosso Do Sul, Mato Grosso, Brazil; ^3^Hartford Hospital Evidence-Based Practice Center, University of Connecticut, Hartford, CT, United States; ^4^Vicerrectorado de Investigacion, Universidad San Ignacio de Loyola, Lima, Peru; ^5^Xiamen Cardiovascular Hospital, Hong Kong, China; ^6^Tianjin Key Laboratory of Ionic-Molecular Function of Cardiovascular Disease, Department of Cardiology, Tianjin Institute of Cardiology, the Second Hospital of Tianjin Medical University, Tianjin, China; ^7^Stroke Service, Neurology Division, Ribeirão Preto Medical School, University of São Paulo, Ribeirão Preto, Brazil; ^8^Department of Medico-Surgical Sciences and Biotechnologies, Sapienza University of Rome, Latina, Italy; ^9^Mediterranea Cardiocentro, Naples, Italy; ^10^Physical Therapy Department, Federal University of Bahia—Universidade Federal Da Bahia, Salvador, Brazil; ^11^Programa de Pós-Graduação em Medicina e Saúde—Universidade Federal Da Bahia, Salvador, Brazil; ^12^Physiotherapy Research Group, UFBA, Salvador, Brazil; ^13^The GREAT Group, Salvador, Brazil; ^14^Division of Cardiovascular Surgery of Pronto Socorro Cardiológico de Pernambuco—PROCAPE, Recife, Brazil; ^15^Department of Surgery, University of Pernambuco—Universidade de Pernambuco, Recife, Brazil; ^16^Nucleus of Postgraduate and Research in Health Sciences of Faculty of Medical Sciences and Biological Sciences Institute—FCM/ICB, Recife, Brazil; ^17^Department of Clinical Research, Federal University of Uberlândia, Uberlândia, Brazil

**Keywords:** stroke, epicardial adipose tissue, systematic review, atherosclerosis, metabolic syndrome

## Abstract

Epicardial adipose tissue (EAT) is correlated with endothelial dysfunction, metabolic syndrome, increased mortality and recent studies showed a possible association with the increased risk of stroke. We performed a systematic review of studies evaluating the association between EAT and stroke. Eighty studies met the inclusion criteria and were consequently analyzed. The review had Five main findings. First, the increased epicardial fat thickness (EFT) may be associated with the stroke episode. Second, regardless of the imaging method (echocardiography, MRI, and CT) this association remains. Third, the association of metabolic syndrome and atrial fibrillation seems to increase the risk of stroke. Fourth, this systematic review was considered as low risk of bias. Despite being unable to establish a clear association between EAT and stroke, we have organized and assessed all the research papers on this topic, analyzing their limitations, suggesting improvements in future pieces of research and pointing out gaps in the literature. Furthermore, the mechanistic links between increased EAT and stroke incidence remains unclear, thus, further research is warranted.

## Introduction

Epicardial adipose tissue (EAT) is more prominent on the right side of the heart on the side wall of the right ventricle and is located between the pericardium and the myocardium (1, 2). It acts not only as anatomical fat deposit, but also as a biologically active tissue that secretes hormones, pro-inflammatory cytokines and proteins (3, 4). The EAT can be determined by imaging tests, such as Computed tomography (CT), Echocardiography (EC) and Magnetic Resonance Imaging (MRI) (5–7).

Determinant factors for increasing EAT are: unbalanced diet, obesity, age ethnicity, gender, low socioeconomic and cultural level, stress and sedentary lifestyle (8–10).Studies have shown that EAT is associated with insulin resistance, diabetes, hypertension, dyslipidemia, atrial fibrillation, increased risk of cardiovascular disease and stroke, the latter being the focus of this study (11–14).

Stroke falls into the category of cerebrovascular diseases that affects the individual due to the change of blood flow to the brain. It is characterized by the acute onset of a neurological deficit, being understood by the rapid occurrence of clinical signs, resulting from focal or global disturbances of brain function, resulting in symptoms lasting longer than 24 h (15, 16).

Findings from the Global Burden of Disease Study (2010) (15) show that the incidence of stroke due to ischemia is 68%, whereas the incidence of hemorrhagic stroke is 32%. A percentage of 87% of strokes due to ischemia, 10 of intracerebral hemorrhage and 3 of subarachnoid hemorrhage were observed, respectively, in the United States (17). According to the World Health Organization (2018) (18, 19), ischemic heart disease and stroke are the largest cause of death in the world, accounting for 15.2 million deaths in 2016. Stroke is the main cause of death in most of Latin American countries, and Brazil has the highest stroke mortality rate in the Americas. The highest incidence occurs after 65 years; however, it is observed occurring earlier in the population (16, 20–22).

Stroke stands out as an important focus of discussion in Public Health with regard to Chronic Non-communicable Diseases, placing a large socioeconomic burden on patients (23). Taking into account that stroke is among the diseases that most affect individuals in various age groups and both sexes worldwide, and that EAT has raised questions about the associated risks, this is the first systematic review and meta-analysis that explores the association of these two factors. In addition, the methods used in systematic reviews seek to minimize biases and improve the reliability and accuracy of conclusions.

## Methods

We performed a systematic review according to a protocol using PRISMA–statement guidelines (24). The review protocol was registered at PROSPERO (registration number: CRD42018091399).

### Search Strategy

A search strategy was developed using the following terms: “*epicardial fat*,” OR “*epicardial adipose tissue*” and “*ischemic stroke*” OR “*brain ischaemia*” OR “*stroke*” as text words and Medical Subject Headings (i.e., MeSH and EMTREE) and searched MEDLINE, EMBASE, Scopus, Cochrane Central Register of Controlled Trials (CENTRAL), Biomed Central, Web of Science, IBECS, LILACS, Congress Abstracts, and Grey literature (Google Scholar and the British Library) for studies published from January 1990 to May 2020. The study was only about articles on human beings and all languages. Reference lists of all available primary studies were reviewed to identify additional relevant citations.

### PECO Strategy

This review included observational studies due to its characteristics of associating a factor and a condition, the construction of the research question was done through the acronym PECO (25) where each letter represents a component of the issue and is presented below:

Population: Individuals of either sexes with 18 years or more;

Exposure and Control: Epicardial adipose tissue measured by Echocardiogram or tomography or MRI.

O: Stroke diagnosed by a physician using CT or MRI.

### Screening of Abstracts for Eligibility

Two reviewers (M.I.R and A.J.G) independently screened the titles and abstracts. The screening process was conducted in Covidence (http://covidence.org). The potentially relevant full texts were read in full, and those that met the inclusion criteria were included in this review. Data from all included studies were also extracted independently by two reviewers and when there was some divergence, a third reviewer resolved the conflict.

### Study Selection

This review included cohort studies (prospective or retrospective), case-control, and cross-sectional studies evaluating the association of epicardial fat and stroke.

#### Inclusion Criteria

Studies with participants of both sexes with 18 years or more;

Populations of patients admitted with stroke.

#### Exclusion Criteria for Studies

We excluded preclinical studies, and studies that evaluated the association with other types of fat.

### Data Extraction

Two reviewers (M.I.R and A.J.G) independently extracted data from the primary studies included in the study. Final decision for inclusion or exclusion of studies in this systematic review was made with reference to the study project registered at PROSPERO. Disagreements about the inclusion or exclusion of the study were resolved by consensus. In the data extraction form we evaluate the data on the characteristics of the patients, methods used, and the outcomes of each study.

### Assessment of Methodological Quality

The quality analysis of the studies was performed by two investigators (E.R.D and M.I.R) independently using the Downs and Black (26) instruments for the case-control and cohort studies and the JBI Critical Appraisal Checklist (27) for cross-sectional studies. Any discrepancies in the evaluation of the quality of studies among researchers were resolved by discussion and consensus among all authors.

### Data Synthesis and Statistical Analysis

The results were described in tables individually; no meta-analysis could be conducted due to substantial heterogeneity of data.

## Results

**Figure 1:**
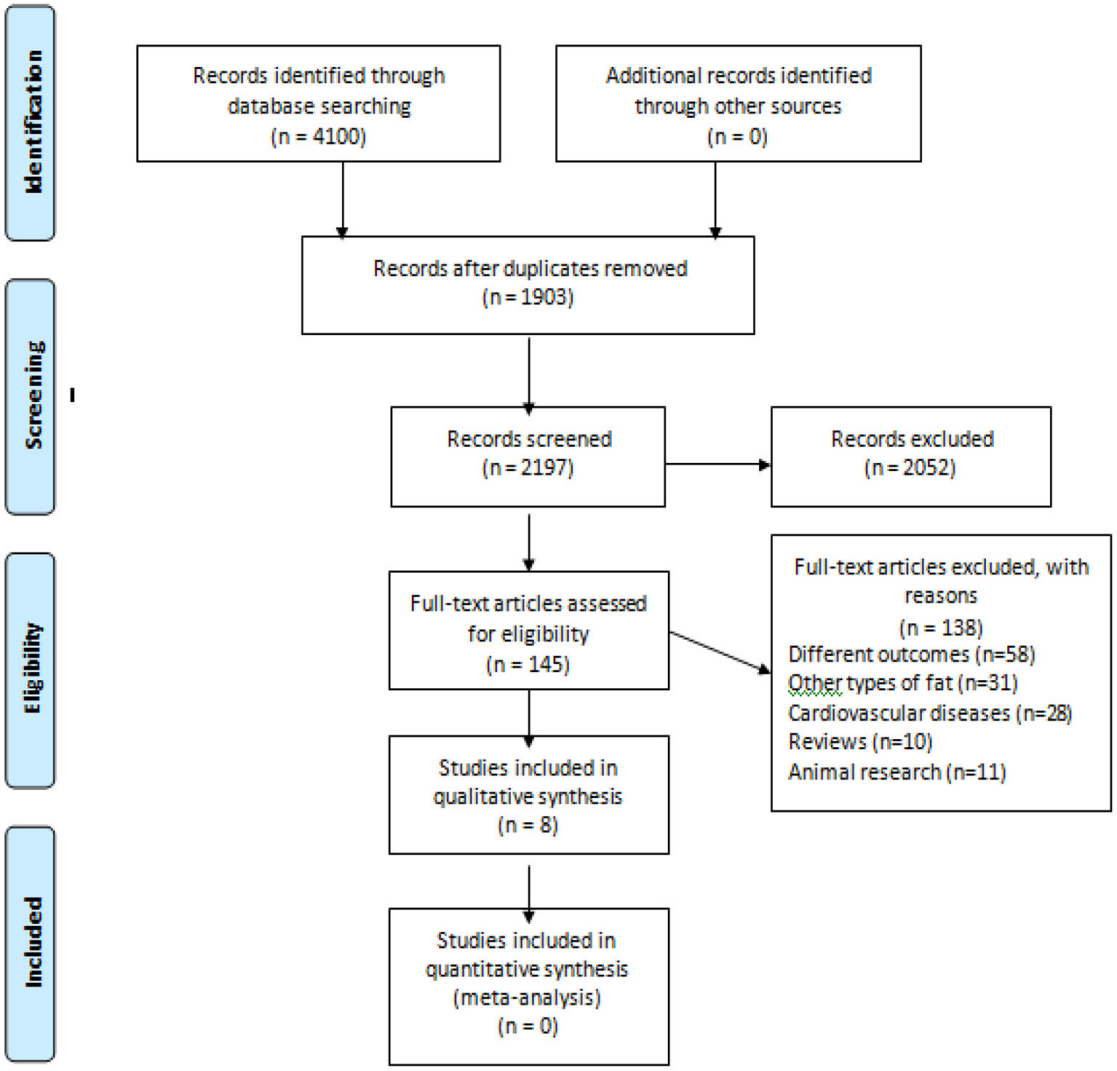
Flowchart.

In the initial search we found 2,197 studies, of which 2,052 were removed after evaluations of titles and abstracts. A total of 145 studies were read in full. From these, 139 studies were excluded because they did not meet the criteria, thus,8 studies were included Gürdal et al. (cross-sectional) (19), Akil et al. (cross-sectional) (28); Altun et al. (case-control) (29); Chu et al. (prospective cohort) (30); Tsao et al. (cross-sectional) (13); Cho et al. (retrospective cohort) (14); Tsao et al. (case-control) (31) and Cosansu and Yilmaz (cross-sectional) (32). The flowchart of the study selection is shown in Figure 1.

These included studies were from countries such as Taiwan (three studies), Turkey (three studies) and Korea (one study). Referring to the sample characterization, the total was 893 individuals with 360 in the control group and 747 in the case group. The elderly population was the most prevalent in the studies (both in the control groups and in the case groups). The age ranges were 50 and 73 years, being the majority composed of females. The characteristics of the included studies are in Table 1.

**Table 1:** Characteristics of primary studies.

**Author/year**	**Country**	**Design**	**Mean age case/control**	**N cases (F/M)**	**N controls (F/M)**	**Definition of EAT and measurement**	**Measurement for stroke**	**Size EAT(cases)**	**Size EAT(controls)**	**Measure of association**
Altun et al. (28)	Turkey	Case-control	71.4 ± 11/68.6 ± 8	61 (34/27)	82 (40/42)	Found between the heart and pericardium/ Echocardiography	Clinical signs in the last 24 h	4,8 ± 0,9mm	3,8 ± 0,7mm	OR 3.178 (95%CI- 1.404–7.195).
Akil et al. (27)	Turkey	Cross sectional	50.5 ± 13.9/53.7 ± 9.0	38 (15/23)	47 (20/27)	Space between the pericardial layers/ Echocardiography	Computed Tomography and magnetic resonance imaging	6,0 ± 1mm	4,4 ± 0,9mm	OR 10.436 (95% CI- 3.032–35.570).
Chu et al. (29)	Taiwan	Prospective cohort	68 ± 10/73 ± 10	93 (30/63)	97 (32/65)	Space located between the visceral pericardium and the external wall of the myocardium/ Echocardiography	Clinical signs	8,1 ± 1,6mm	4.4 ± 0.9mm	HR univariable 1.286 (95% CI - 1.168–1.417). HR multivariable 1.211 (95% CI - 1.084–1.351).
Tsao et al. (13)	Taiwan	Cross sectional	64.11 ± 11.43/63.25 ± 7.56	27 (8/21) Patients with atrial fibrillation	20 (5/15)	Fat located between the visceral pericardium and the myocardium / Computed Tomography	Clinical signs	53,07 ± 14.67 cm3	21.46 ± 14.64 cm3	Univariable OR 1,15 (95% CI-1,09–1,21). Multivariable 1,12 (95% CI - 1,06–1,19).
Cho et al. (14)	Korea	Retrospective Cohort	65.4 ± 12.1/75.0 ± 10.6	Ischemic stroke without AF: 179 (65/114) ischemic stroke with AF: 35 (20/15)	There was no control	Epicardial fat was defined as the echo-free space between the Outer wall of the myocardium and the visceral layer of t e pericardium. Echocardiography	Magnetic resonance imaging	6.5 ± 1.2mm	5.3 ± 1.2mm	Uninformed.
Tsao et al. (30)	Taiwan	Case-control	uninformed	20 Patients with atrial fibrillation	34	uninformed/Computed Tomography	uninformed	60.27 ± 13.10 cm3	24.34 ± 6.78 cm3	Uninformed.
Gürdal et al. (18)	Turkey	Case-control	43 ± 8/38 ± 7	40 (16/24)	37 (20/17)	Epicardial adipose tissue (EAT), is a visceral fat depot of the heart, is located on the surface of the heart between the myocardium and visceral pericardium/ Echocardiographic	ESUS was defined according to the criteria proposed by the Cryptogenic Stroke/ESUS International Working Group as a visualized nonlacunar brain infarct in the absence of the following causes: extracranial or intracranial atherosclerosis causing ≥50% luminal stenosis in arteries supplying the area of ischemia; major cardioembolic sources; and any other specific cause of stroke (eg, vasculitis, dissection, migraine/vasospasm, drug misuse)./MRI	5.51 ± 0.82mm	3.96 ± 0.51	Uninformed
Cosansu and Yilmaz (31)	Turkey	cross-sectional	75.19 ± 9.39; 73.72 ± 8.60	80 (50/30)	80 (46/34)	Echocardiographic evaluations were performed in the first three days after hospitalization.	The prediction of acute ischemic stroke by echocardiographic EFT was estimated.	5.90 ± 1.35 mm	8.55 ± 1.08 mm	OR Univariate analysis: EFT = 23.449, 95%CI: 5.773–95.244 (*p* < 0.0001); OR Multivariate analysis: EFT = 7.356, 95%CI: 3.880–13.947 (*p* < 0.0001)

*CI, confidence interval; AF, atrial fibrillation; OR, odds ratio; HR, hazard ratio*.

Akil et al. (28) gathered population of 85 individuals, 38 patients belonging to the case group, a BMI of 25.1 ± 1.3 kg/m^2^, and 47 patients in the control group, with a BMI of 24.9 ± 4.7 kg/m^2^. The cut-off point established by the author for epicardial fat in order to demonstrate risk was 5.35 mm, with a sensitivity of 73.7 and 83.6% of specificity, and the ROC curve (95% CI) provided a value of 0.802 (0.699–0.905)—*p* > 0.001. The validation of the patients with ischemic stroke was given by Magnetic Resonance and Computed Tomography. The findings demonstrate a significant increase in epicardial adipose tissue (5.95 ± 1.14 mm) in patients with ischemic stroke compared to patients in the control group (without ischemic stroke) (4.86 ± 0.68 mm)—*p* < 0.001.

Altun et al. (29) gathered a sample of 143 patients, in which the case group the n was 61 with a mean age of 71.4 ± 11 years, 56% female with a waist size evaluated at 100.8 cm. In the control group, the n was 82 with a mean age of 68.6 ± 8 years, where the male predominated (51%), with a waist circumference of 100.7 cm. The method used to determine the epicardial adipose tissue was also through echocardiography. The determination of the ischemic stroke was defined by the clinical signs of focal disturbance of the cerebral function, of probable ischemic origin, with duration of more than 24 h. However, a cranial computed tomography scan was performed in 100% of patients and an MRI in 84% of the patients and the National Institute of Health Stroke Scale (NIHSS) score was also used to assess the severity of stroke. With a cutoff point of 4.28 mm for epicardial adipose tissue [ROC curve = 0.84 (95% CI 0.772–0.908)], indicating a sensitivity of 81% and specificity of 81% (*p* = < 0.001), the correlation of epicardial adipose tissue and ischemic stroke showed that in the case group it was higher (4.8 ± 0.9 mm) than in the control group (3.8 ± 0.7 mm)—*p* < 0.001.

Tsao et al. (13) studied 115 patients divided into three groups: a group (case) with 27 patients with atrial fibrillation (AF) and stroke [Body mass índex (BMI) of 23.89 kg/m^2^ ± 3.51], another group consisted of 68 with atrial fibrillation, but no stroke and one last group (control) with 20 patients without atrial fibrillation and without stroke (BMI of 24.97 kg/m^2^ ± 3.14). The method used to detect stroke was through clinical signs and the measurement of epicardial adipose tissue was performed by means of a Computed Tomography scan with a cutoff point of 40.68 cm3, where the result given to the case group (stroke + AF) was 53.07 ± 14.67 cm3 and for the control group (without AF and without stroke) was 21.46 ± 14.64 cm3 (*p* < 0.001). However, the group with AF without stroke had a score of 29.85 cm3 in its epicardial adipose tissue value. In relation to additional biochemical tests, Tsao et al. (13) presented LDL values of 103.85 ± 25.06 mg/dl for the case group and 105.17 ± 19.94 mg/dl for the control group.

The study by Tsao et al. (31) included three groups with individuals who had atrial fibrillation and had the epicardial adipose tissue measured in the left atrium. Although it was not possible to obtain the article completely, its published abstract provided enough data for it to be included in this review. The sample consisted of 122 individuals, 34 in the control group (group 1), 68 patients in the group with AF and no history of stroke (group 2) and 20 patients in the group with stroke and AF (group 3). The epicardial adipose tissue measurement was performed by computed tomography and the control group obtained a value of 24.34 ± 6.78 cm3 in relation to the group with stroke and AF that reached a value of 60.27 ± 13.10 cm3. The value of group 2 (patients with AF and without stroke) obtained a value of 32.11 ± 11.87 (*p* > 0.001).

Chu et al. (30) obtained 32 women and 65 men in the control group, being the BMI 26.8 ± 4.3 kg/m^2^. In the experimental group, 30 women and 63 men, with a BMI of 25.6 ± 3.9 kg/m^2^, totaled a n of 190.This study through a ROC curve found that the best value between EAT and prediction of cardiovascular disease events is 6.0 mm, where the value ≤ 6.0 mm was attributed to the control population and a value ≥ 6.0 mm for the case group. Therefore, the control group, when submitted to Echocardiography to measure the epicardial adipose tissue, obtained a value of 4.4 ± 0.9 mm when compared to the group that had a finding of 8.1 ± 1.6 mm (*p* <0.001).

Gürdal et al. (19) investigated the measurement of echocardiographic EAT in young patients with embolic stroke of undetermined source (ESUS) Patients with ESUS had a significantly higher EFT than the control group (5.51 ± 0.82 vs. 3.96 ± 0.51; *P* < 0.01). In addition, there was a positive correlation between EFT and serum C-reactive protein levels (*r* = 0.284; *P* < 0.05). As an ideal cutoff, a high-risk EFT value of 4.6 mm was determined to predict ESUS, with a sensitivity of 87.5% and a specificity of 81.1%. EAT was significantly higher in young patients with ESUS than in healthy subjects.

Cho et al. (14) obtained a population composed of 214 patients diagnosed with ischemic stroke by magnetic resonance imaging, whose mean age was 66.8 ± 12.3 years and 39.7% of the sample was represented by women. The study population was divided into two groups, a group that had ischemic stroke + AF (*n* = 35) (BMI of 24.3 ± 3.3 kg/m^2^) and one group with ischemic stroke without AF (*n* = 179) 3.2 kg/m^2^). The determination of the EAT was performed by means of an Echocardiography, which showed that the case group (with ischemic stroke + AF) presented values of 6.5 ± 1.2 mm vs. 5.3 ± 1.2 mm of the ischemic stroke group without AF (control group), (*p* < 0.001). Additional examinations included dyslipidemia in 31.3% of the population with (AF without stroke) and 22.9% in the population with AF).

Cosansu and Yilmaz (32) included 80 AF patients with acute ischemic stroke (AIS) and 80 AF controls with the same age and sex without AIS, in this study it was observed that compared to the control group, patients with AF with AIS had significantly greater epicardial thickness (8.55 ± 1.08 vs. 5.90 ± 1.35 mm; *P* < 0.0001), and in multivariate regression analysis indicated that EFT independently predicts AIS in patients with AF.

### Risk of Bias of the Included Studies

Using the Downs and Black tool, methodological evaluation showed an average of 20 points, being the methodological quality of the studies considered good, described in Table 2.

**Table 2:** Quality of included studies evaluated with the downs and black checklist (studies with case-control design).

**References**	**1**	**2**	**3**	**4**	**5**	**6**	**7**	**8**	**9**	**10**	**11**	**12**	**13**	**14**	**15**	**16**	**17**	**18**	**19**	**20**	**21**	**22**	**23**	**24**	**25**	**26**	**27**	**Total**
Altun et al. (28)	1	1	1	1	0	1	1	0	1	1	1	1	1	0	0	0	0	1	1	1	1	1	0	0	0	0	5	21
Tsao et al. (30)	1	1	0	1	0	1	0	0	0	1	0	0	0	0	0	0	0	0	1	1	0	0	0	0	0	0	4	11
Chu et al. (29)	1	1	1	1	0	1	1	0	1	1	0	0	0	0	0	1	1	1	1	1	0	1	0	0	0	1	5	20
Cho et al. (14)	1	1	1	1	1	1	1	0	1	1	1	1	1	1	1	0	1	1	1	1	1	1	0	0	0	1	5	26
Gürdal et al. (18)	1	1	1	1	1	1	1	0	0	1	1	1	1	1	0	0	0	1	1	1	1	1	0	0	0	0	5	22

The JBI Critical Appraisal Checklist, although not providing a score at the end of the evaluation, also resulted in a good methodological quality of the post-evaluation studies shown in Table 3. After analyzing the quality of the studies, this systematic review was considered as low risk of bias.

**Table 3:** Quality of included studies assessed by the *JBI critical appraisal checklist (cross sectional studies)*.

**Criteria**	**References**		
	**Tsao et al. (13)**	**Akil et al. (27)**	**Cosansu and Yilmaz (31)**
1. Were the criteria for inclusion in the sample clearly defined?	Yes	Yes	Yes
2. Were the study subjects and the setting described in detail?	Yes	Yes	Yes
3. Was the exposure measured in a valid and reliable way?	Yes	Yes	Yes
4. Were objective, standard criteria used for measurement of the condition?	Yes	Yes	Yes
5. Were confounding factors identified?	Yes	Yes	Yes
6. Were strategies to deal with confounding factors stated?	Yes	Yes	Yes
7. Were the outcomes measured in a valid and reliable way?	Yes	Yes	Yes
8. Was appropriate statistical analysis used?	Yes	Yes	Yes

## Discussion

This review included eight studies in which it can observed an association between EAT and stroke involvement. Studies showed an association of increased thickness of epicardial fat measured by methods such as echocardiography, MRI, and CT with stroke diagnosed by clinical examination, MRI, or CT. Different methods of quantifying epicardial fat suggest that its increased thickness is associated with an increased risk of the patient having a stroke. Obesity and central adiposity are associated with the increase in risk factors for atherosclerosis and fibrillation and, consequently, with a greater incidence of stroke in this population. Ischemic stroke in the studies had a greater relationship between the increase in epicardial fat, central adiposity and risk factors. The percentage of dyslipidemia was higher in the stroke group. In patients with high volume and/or thickness of epicardial fat, they had a greater number of risk factors and their associated stroke.

The included studies established an association with older age and BMI with the thickness of the EAT. The relationship between BMI and EAT was also observed by Iacobellis et al. (33), where individuals with metabolic syndrome (MetS) with a waist ratio of 104 cm in men and 98 cm in women had significantly higher values of EAT (9.5mm ± 7–20 in men) and 4.5 mm ± 3–5.5 in women when compared to individuals without MetS (92 cm in men and 82 cm in women) with an EAT of 7.5 mm ± 6–15 in men and 3.5 mm ± 2–4.5 in women—*p* < 0.001.

In addition to obesity, another possible link between stroke and increased EAT is inflammation. Mean of neutrophil/lymphocyte ratio (NLR) was significantly greater among ischemic stroke patients in relation to the control group (2.5 ±.6 vs. 1.8 ± 0.4, *P* < 0.001). (28).

Altun et al. (29) demonstrated in their findings that individuals who suffered a stroke event had less aortic distensibility compared to the control group, corroborating these findings, Dogan et al. (34) observed that in patients with newly diagnosed hypertension, the increase in EAT was significantly related to impaired aortic elastic properties.

The study by Tsao et al. (31) evaluated the volume of EAT around the left atrium and its association with the risk of stroke in patients with atrial fibrillation (AF). case group (stroke + AF) than in the control group (without AF and without stroke). In this study, it can be seen that the accumulation of EAT in the left atrium is directly related to patients who suffered stroke and who had atrial fibrillation. This fact corroborates the evidence published by Batal et al. (35), who reported that greater thickness of the posterior left atrium is associated with AF. It was also demonstrated by Tsao et al. (36) in a sample of patients with AF and control patients that the EAT was significantly increased in patients with AF and was associated with a higher incidence of AF recurrence after catheter ablation. In addition, a meta-analysis carried out also showed an increase in EAT in patients with AF (37).

Chu et al. (30) demonstrated that higher volumes of EAT are associated with future risks of cardiovascular disease (including stroke) in patients with atrial fibrillation in an elderly population (mean of 70 years), and it was independent association between age and EAT was observed. Other studies (8, 32, 38) also observed this finding and showed that the volume of the EAT tends to increase with age, and the study by Abbara et al. (39) points out that the EAT is thicker in people over 65 years old. In addition, the population studied by Chu et al. (30) presented overweight BMI in both groups, enhancing what detailed studies previously showed that overweight may be influencing the thickness of epicardial fat.

Cho et al. (14) conducted a retrospective cohort to assess the thickness of the EAT and the levels of free fatty acids as predictors of acute ischemic stroke in patients with AF. The analysis of the EAT was performed by means of echocardiography, which showed by means of multivariate logistic regression, the study demonstrated that age and EAT are independently associated with ischemic stroke.

Mahabadi et al. (40) included a total of 1,267 participants who underwent computed tomography to quantify the volumes of EAT, intrathoracic fat and visceral abdominal adipose tissue. In this study it was shown that EAT was significantly associated with coronary artery disease and acute myocardial infarction, however, only visceral abdominal adipose tissue was associated with stroke. The lack of association between EAT and stroke in this study can be explained by the small number of cerebrovascular events that the studied population had (in comparison with other cardiovascular diseases, such as acute myocardial infarction).

Taguchi et al. (41) observed a significant association between the volume of EAT and the prevalence of coronary artery disease in their sample of Japanese men. Cosansu and Yilmaz (32) observed in their study that EFT can be an independent predictor for the development of acute ischemic stroke in patients with AF.

We must consider the possible role of epicardial fat thickness in strokes of unknown cause (cryptogenic strokes, also including ESUS). The association between different types of pathogenic strokes and the thickness of epicardial fat may be related to the presence of atrial heart disease, which is currently considered one of the possible hidden causes of this type of stroke. In this view, multiple factors (such as inflammation, thickness of epicardial fat, changes in the autonomic nervous system) may be linked to atrial changes that favor the cardioembolic mechanisms in these strokes (41–45).

## Strengths and Limitation of the Study

Firstly, we observed that there are some differences regarding the EAT cut-off points established among authors or even the lack of it in some studies. Second, a meta-analysis could not be carried out showing significant difference in study designs and missing information in group was also a factor of decreased the quality of the studies. We can also highlight a moderate heterogeneity between studies, and different imaging modalities such as echocardiography measuring thickness and mm, and CT and MRI measuring epicardial fat diameter, the small number of participants in some studies and the lack of control group was also a factor of decreased the quality of the studies. A TOAST classification denotes five subtypes of ischemic stroke: (1) atherosclerosis of large arteries, (2) cardioembolism, (3) occlusion of small vessels, (4) stroke of another given etiology and (5) stroke of undetermined etiology. Unfortunately, this classification hás not been obtained in several studies. In most studies, EFT was considered a risk factor for stroke and in only one cross-sectional study as a predictor of stroke. These data should be corroborated in large outlined studies and with a longer follow-up time. In this first systematic review to analyze the association between EAT and stroke, it was seen that the greater thickness of the EAT was more prevalent in all stroke groups than in groups without stroke. In summary of the data found, five studies used echocardiography to measure the thickness of epicardial fat and three performed CT and/or MRI to assess its volume. Patients with higher BMI, waist circumference, were older, obese, embolic stroke (ESUS), stroke and AF, respectively, had higher levels of epicardial fat. In the assessment of epicardial fat thickness, a greater association was observed with the NT-proBNP biomarkers and when it was evaluated by volume, an association was found with the increase in FFA levels (46–53).

## Conclusion

Despite not being able to establish an association between EAT and stroke, we have organized and assessed all the research papers on this topic, highlighting their limitations, suggesting targets for future research and pointing out gaps in the literature.

## Data Availability Statement

The raw data supporting the conclusions of this article will be made available by the authors, without undue reservation.

## Author Contributions

LR, ED, GB-Z, and LDL conceived the study idea and planned the study methodology. LR, GB-Z, TL, GT, OP-N, AD, MN, MS, ER, and AH participated in the design and coordination of the study. LR was primarily responsible for protocol writing and developed the search strategy. AG and MR screened the studies, conduct data extraction and analysed the review findings. All authors read the drafts, provided comments and agreed on the final version of the manuscript.

## Conflict of Interest

The authors declare that the research was conducted in the absence of any commercial or financial relationships that could be construed as a potential conflict of interest.

## Abbreviations

EAT: epicardial adipose tissue
EFT, epicardial fat thickness, CT, computed tomography, EC: echocardiography
MRI, magnetic resonance imaging, AF: atrial fibrillation
BMI, body mass index, ESUS: embolic stroke of undetermined source
AIS: acute ischemic stroke
NIHSS: National Institute of Health Stroke Scale.
